# Nano-Enabled Precision Management of Plant Anthracnose: Mechanisms of Action, Application Advances, and Future Perspectives

**DOI:** 10.3390/jof12080615

**Published:** 2026-08-16

**Authors:** Shuo Miao, Chaoqiong Liang, Xinghong Wang

**Affiliations:** 1 Experimental Centre of Forestry in North China, Chinese Academy of Forestry, Beijing 102300, China; ms@caf.ac.cn; 2 Shaanxi Academy of Forestry, Xi’an 710016, China

**Keywords:** plant anthracnose, *Colletotrichum* spp., nanomaterials, nano-delivery systems, antifungal mechanisms, green control

## Abstract

Plant anthracnose, caused by *Colletotrichum* spp., is a class of significant diseases that severely threatens global crop production. Traditional management strategies, including chemical control, are hindered by inherent limitations such as the frequent emergence of pathogen resistance, low pesticide utilization efficiency, high environmental residue risks, and inconsistent field efficacy. Moreover, achieving further improvements in control efficacy is constrained by the complex biological characteristics of *Colletotrichum* species, particularly their hemibiotrophic lifestyle and latent infection strategies. In recent years, nanotechnology has emerged as a potential approach for the management of anthracnose. This review summarizes the research progress of various nanomaterials in the control of plant anthracnose. It analyzes the proposed multi-mechanism modes of action of nanomaterials tailored to the specific infection traits of *Colletotrichum*. Particular emphasis is placed on analyzing the theoretical mechanisms and preliminary in vitro evidence of nano-enabled controlled-release systems in addressing asymptomatic latent infections and achieving targeted, precise delivery. However, it must be emphasized that most current findings are derived from laboratory or controlled-environment studies, and the actual field-scale effectiveness, long-term environmental behavior, and multi-trophic safety of many nanomaterials remain insufficiently confirmed. Building upon these insights, this review thoroughly evaluates the critical challenges facing the agricultural field application of nanomaterials, such as ecotoxicity, formulation stability, scalable industrial production, and regulatory gaps. This review aims to provide objective theoretical support and technical references for achieving safe, efficient, and sustainable nano-enabled green management of plant anthracnose.

## 1. Introduction

Plant anthracnose is a major disease caused by fungi of the genus *Colletotrichum* spp. Ranking among the top ten most important plant pathogenic fungi globally, the genus *Colletotrichum* possesses a broad host range, infecting hundreds of critical economic crops across families such as Poaceae, Solanaceae, Rosaceae, and Cucurbitaceae [1]. In major strawberry-producing regions like North America, anthracnose can induce root rot, crown rot, leaf spot, and fruit rot, leading to yield losses of up to 70% in severe cases. It is widely recognized as a primary disease limiting the sustainable development of the industry [2]. In mango-growing areas, pre- and post-harvest anthracnose caused by the *Colletotrichum gloeosporioides* species complex is a primary cause of fruit rot and the decline in commercial value, potentially resulting in up to 100% yield loss under humid conditions [3,4]. Pepper anthracnose, triggered by *C. capsici* and *C. gloeosporioides* infections, leads to fruit decay and malformation, severely compromising both yield and quality [5]. Furthermore, in citrus-producing regions worldwide—including Italy, Portugal, and China—anthracnose has become a critical limiting factor affecting the post-harvest quality and shelf life of fruits such as sweet oranges [1]. Frequent outbreaks of anthracnose stalk rot in maize, *Camellia oleifera* anthracnose, and anthracnose in the medicinal plant *Polygonatum cyrtonema* have also imposed a heavy burden on agricultural production [6,7,8,9].

*Colletotrichum* species exhibit profound host adaptability and genetic variability, leading to the formation of differentiated dominant pathogenic populations across various crop-producing regions. In Chinese provinces such as Zhejiang, Shanghai, and Fujian, the pathogenic populations of strawberry anthracnose are primarily composed of *C. siamense*, *C. fructicola*, and *C. aenigma*. Among these, *C. siamense* exhibits the strongest pathogenicity and displays distinct physiological race differentiation across different geographical territories [10,11,12]. The genetic diversity and variations in pathogenicity of the genus *Colletotrichum* are evident globally; for instance, the dominant species in Mexican mango-producing areas are *C. siamense* and *C. asianum* [3], whereas anthracnose in South Korean apple orchards is mainly driven by *C. fructicola* and *C. siamense* [1]. Because *Colletotrichum* can survive on a diverse array of host plants and plant debris and disseminate over long distances via wind, rain, seedlings, and agricultural practices, the disease is difficult to eradicate once established, posing a continuous threat to global food security and agricultural trade [13,14].

Over the years, the management of plant anthracnose has predominantly relied on chemical fungicides, cultural practices, and biological control. In chemical control, benzimidazoles (MBCs, e.g., carbendazim and thiophanate-methyl), prochloraz, tebuconazole and fluazinam are currently the primary agents utilized in the field [8,12,15]. However, due to the high population genetic diversity and rapid reproductive rate of *Colletotrichum* species, they are highly prone to developing fungicide resistance under continuous selection pressure. Studies indicate that point mutations in the β-tubulin gene reduce the binding affinity of benzimidazole fungicides to their target proteins, leading to the emergence of highly resistant strains across various *Colletotrichum* species [16,17]. Furthermore, the resistance risk associated with QoI (quinone outside inhibitors) fungicides, such as azoxystrobin—which target mitochondrial respiratory chain complex III—cannot be ignored; widespread QoI resistance has already been observed in strawberry anthracnose populations [18,19]. Additionally, the pesticide utilization efficiency of conventional formulations is typically only 10%. A substantial volume of active ingredients enters soil and water bodies via leaching, runoff, and volatilization, resulting in environmental pollution and ecotoxicity [5].

Biological control, as an alternative strategy, can effectively circumvent the resistance risks and ecological damage associated with chemical pesticides. Its mechanisms encompass competition, parasitism, the production of antimicrobial substances, and the induction of plant resistance [20]. For example, the endospores of *Bacillus velezensis* strain S26 exhibit significant inhibitory effects against seven pathogens causing grape anthracnose by competing for ecological niches and nutritional space [21]. Furthermore, *B. amyloliquefaciens*, *B. subtilis*, *Trichoderma harzianum*, and various marine yeasts have demonstrated robust control efficacy against different *Colletotrichum* species complexes [20]. However, the field efficacy of biological control is frequently constrained by environmental variables such as temperature, ultraviolet radiation, humidity, and competition from indigenous microbiota. The colonization stability and persistence of certain antagonistic microbes in the field still require improvement, making it challenging for a single biological control method to fully satisfy the demands of large-scale agricultural management [21]. Cultural practices, serving as the foundational approach for source management, can establish a synergistic and complementary control system with biological methods by severing pathogen transmission chains and reducing the initial inoculum load in the field. Although agronomic measures—such as the thorough removal of diseased residues, rational crop rotation, and the cultivation of disease-resistant varieties—can mitigate disease occurrence to some extent, relying solely on these practices is often insufficient to achieve economically viable disease control due to the latent infection characteristics and broad host range of *Colletotrichum* species. *Colletotrichum* can infect hosts during the flowering or young fruit stages and remain in an asymptomatic latent state within host tissues for extended periods, only triggering severe disease outbreaks upon fruit ripening or under favorable environmental conditions. This biological trait complicates disease monitoring and management [1,14,22]. Therefore, it is imperative to integrate the technical advantages of chemical, biological, and agricultural practices to develop novel control technologies that are highly efficient, low in toxicity, environmentally friendly, and less likely to induce pathogen resistance.

Given the limitations of existing disease control strategies, nanotechnology offers a promising research direction to overcome traditional technical bottlenecks. Compared to conventional pesticide formulations, nanomaterials—characterized by their high specific surface areas, tunable surface chemical properties, drug-loading capacities, and physical antibacterial activities—have shown potential to achieve multiple objectives, including improved delivery, sustained release, enhanced bioactivity, and reduced environmental residues. More crucially, nanotechnology presents the possibility of multi-mechanism synergistic actions tailored against the complex and dynamic infection strategies of *Colletotrichum*. These strategies include high-turgor mechanical penetration by appressoria, the transition between intracellular biotrophic and necrotrophic stages, the manipulation of host hormone signaling (SA/JA/ABA/ET), and precise regulatory networks governed by transcription factors (e.g., CgrFlbC, CsCreA) and MAPK signaling pathways [14,23,24,25,26,27]. For instance, nanomaterials can theoretically disrupt the pathogen’s physical penetration capabilities by blocking appressorial melanization or compromising cell membrane integrity. Simultaneously, they can serve as nanocarriers to deliver double-stranded RNA (dsRNA), achieving gene silencing of key virulence factors (e.g., *CgCOM1*) to weaken the pathogen’s virulence at the genetic level [28,29]. Furthermore, targeting the oxidative stress defense systems induced by *Colletotrichum*, nanoparticles with enzyme-like activities (nanozymes) can continuously catalyze the overproduction of reactive oxygen species (ROS), thereby overwhelming the fungal antioxidant defenses [30]. However, a successful transition from laboratory efficacy to field-scale adoption hinges on resolving practical bottlenecks, including potential ecotoxicity, production scalability, and the stability of nano-formulations under open-field conditions. To address these issues, this review provides a systematic and objective analysis of the progress and constraints of nano-enabled approaches, while outlining future paths—such as “safe-by-design” smart-responsive systems—to foster the precise and green management of anthracnose.

## 2. Infection Strategies of *Colletotrichum* Species

**Figure 1:**
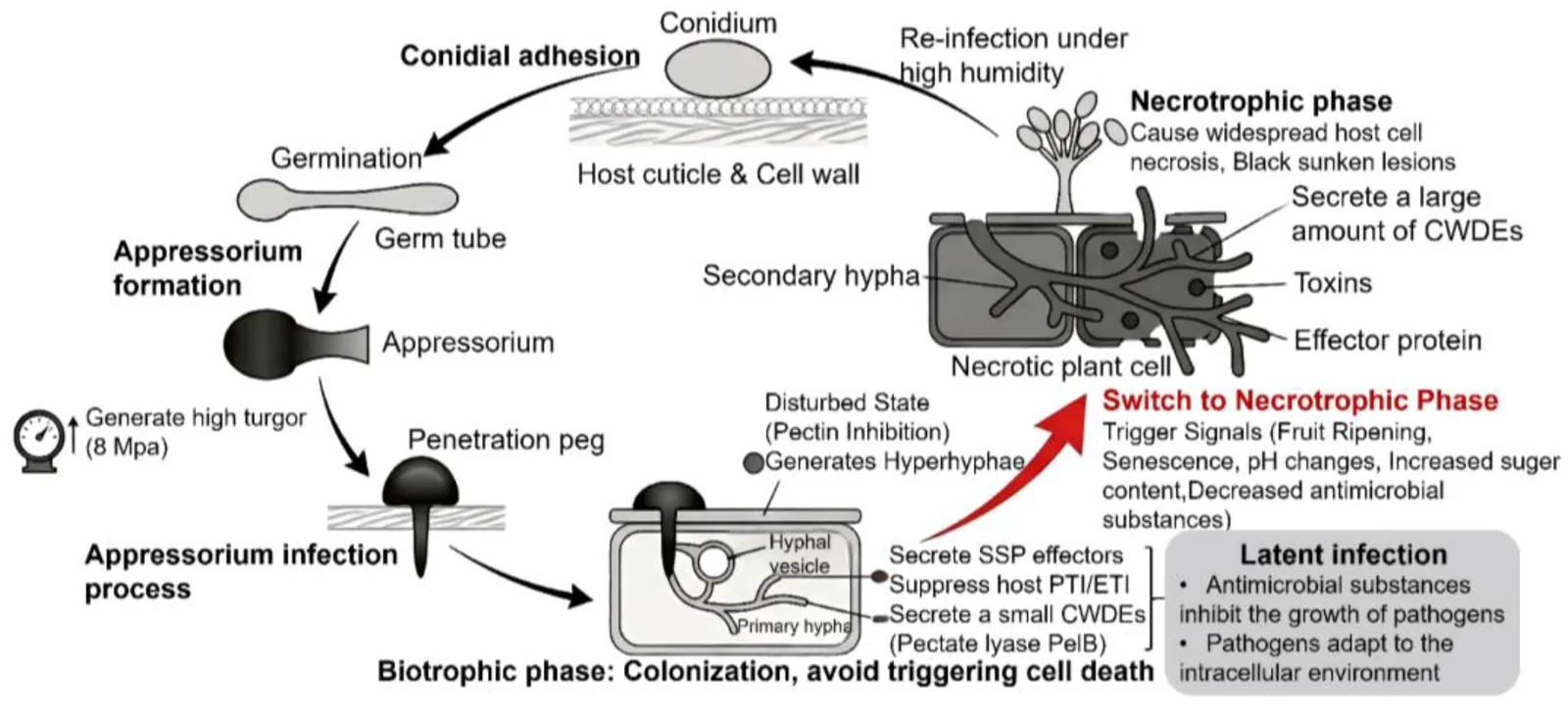
The life cycle and infection strategies of *Colletotrichum*.

The successful infection of *Colletotrichum* species depends on the development of specialized infection structures and a complex network of pathogenic factors. Its life cycle is divided into asexual and sexual stages, with the asexual stage serving as the primary driver of disease epidemics in the field. This pathogen exhibits a classic hemibiotrophic infection strategy; the infection process initiates with a transient, asymptomatic biotrophic phase and subsequently transitions into a destructive necrotrophic phase, ultimately culminating in tissue rot and the appearance of characteristic lesions (Figure 1).

During the initial stages of infection, conidia adhere to the plant surface and rapidly germinate to form germ tubes under favorable temperature and humidity conditions. Upon perceiving host signals, the tip of the germ tube differentiates into a highly specialized infection structure known as the appressorium. Melanin deposition within the appressorial cell wall is crucial for the generation of high turgor pressure. Accompanied by the accumulation of glycerol and other osmotically active substances (osmolytes), an enormous turgor pressure of up to 8 MPa can be generated within the appressorium. This mechanical pressure is converted into a powerful penetrative force, driving the penetration peg to breach the host cuticle and cell wall directly, thereby accomplishing primary infection [1]. It should be explicitly stated, however, that the rate of infection development, tissue colonization speed, and symptom structures vary significantly among different *Colletotrichum* species, host cultivars, and varying macro- and microclimatic conditions. For instance, while the chronological infection of *C. siamense* CA17 on *Camellia oleifera* leaves illustrates a general model (with conidia germinating at 4 hpi, forming melanized appressoria by 6 hpi, colonizing near the epidermis at 24 hpi, and expanding extensively by 72 hpi) [9], other species under sub-optimal temperatures or on alternative hosts can remain in the pre-penetration stage for several days. At the molecular level, appressorium formation is accompanied by large-scale transcriptional reprogramming. For instance, during the appressorium formation stage of *C. gloeosporioides*, more than 4000 genes are upregulated, 468 of which are appressorium-specific and involved in pathways such as secondary metabolism and molecular transport [31]. The precise regulation of these genes constitutes the molecular foundation of appressorium maturation. For example, the C2H2-type transcription factor CgrFlbC in *C. graminicola* participates in regulating hyphal growth, cell wall integrity, oxidative stress responses, and hyphopodia formation; its deletion mutant fails to form hyphopodia, resulting in a significant reduction in virulence [24]. Understanding these specific fungal life stages is critical for the development of targeted nano-enabled intervention strategies, linking structural vulnerabilities directly to nanoparticle selection.

Following the breach of the cell wall by the penetration peg, certain species develop primary hyphae that grow biotrophically within living host cells, thereby avoiding the induction of host cell death [1]. Morphologically, upon entering host cells, some species, such as *C. higginsianum*, form a biotrophic vesicle that subsequently expands into thick primary hyphae [32]. During this phase, small secreted protein (SSP) effectors produced by the pathogen play a pivotal role in establishing and maintaining the biotrophic stage. These effector proteins effectively suppress early host defense responses, including pattern-triggered immunity (PTI) and effector-triggered immunity (ETI), allowing the pathogen to stably colonize during this transient period of interaction with living host cells [32]. Another critical factor involves the host-specific toxins and cell wall-degrading enzymes (CWDEs) secreted by *Colletotrichum*, with the latter encompassing pectinases, cellulases, and hemicellulases that break down plant cell wall components and facilitate intercellular and intracellular hyphal expansion [1]. For instance, the pectin lyase PelB secreted by *C. gloeosporioides* is induced during the biotrophic phase, initiating the degradation of the host cell wall middle lamella to pave the way for the impending necrotrophic phase [33].

Notably, *Colletotrichum* species widely exhibit latent infection characteristics, wherein the pathogen infects host tissues during the flowering or young fruit stages without eliciting symptoms. The pathogenic phenotype surfaces only upon fruit ripening or alterations in the host’s physiological state, triggering post-harvest disease outbreaks and complicating disease monitoring and management [1,14]. Molecular studies indicate that this quiescent or “latent” state represents a delicate equilibrium between host defenses and pathogenic mechanisms. On one hand, high concentrations of antimicrobial compounds (such as polyphenols, tannins, and phytoalexins) and active defense responses in immature fruits suppress pathogen growth [20]. On the other hand, the pathogen itself undergoes physiological adjustments to adapt to the intracellular environment; for example, a P450 monooxygenase, CgPepCYP, expressed in *C. gloeosporioides*, has been shown to be essential for its colonization [34].

At the biochemical level, host ripening events act as chemical switches instructing *Colletotrichum* to transition from latency to necrotrophy. During ripening, host microenvironmental alterations—such as pH shifts, tissue softening, and sudden sugar accumulation—reprogram fungal gene expression. Specifically, pH shifts activate the PacC transcription factor pathway to induce massive pectate lyase secretion, while sugar spikes repress carbon catabolite genes and activate virulence-associated G-protein and cAMP-MAPK cascades. Consequently, the pathogen transitions to an active necrotrophic phase, producing abundant secondary hyphae for rapid colonization. During this phase, the pathogen produces abundant secondary hyphae that rapidly extend both intercellularly and intracellularly. It secretes large quantities of CWDEs (such as polygalacturonases and pectin lyases), toxins, and effector proteins, triggering widespread host cell necrosis and characteristic dark, sunken lesions, which ultimately culminate in post-harvest disease outbreaks [1,20]. Research has demonstrated that *C. acutatum* can form appressoria and secondary conidia on asymptomatic leaves, serving as a source of inoculum for continuous reinfection in the field, which is particularly critical in high-humidity environments such as greenhouses and nurseries [35].

Regarding the molecular regulatory networks underlying pathogenesis, the virulence of *Colletotrichum* is regulated by multi-layered and multiplexed signaling pathways. For example, the knockout mutant of the C2H2-type transcription factor CsCreA in *C. siamense* exhibits only 55% of the wild-type virulence on rubber leaves [23], while the MAPK kinase gene *Cgl-SLT2* and the ABC transporter gene *CgABCF2* have both been proven essential for appressorium formation and pathogenicity [26,27]. During the molecular interaction with hosts, the hemibiotrophic nature of *Colletotrichum* enables it to interfere with multiple host hormone signaling pathways to facilitate infection. Plants typically rely on the salicylic acid (SA) pathway to defend against biotrophic pathogens and the jasmonic acid/ethylene (JA/ET) pathways to combat necrotrophic pathogens [25]. However, *C. gloeosporioides* can manipulate host signaling via in vitro ethylene production to accelerate fruit ripening and tissue softening, thereby creating favorable conditions for the necrotrophic phase [36,37]. Although *C. acutatum* infection partially activates the SA and JA defense pathways in strawberry, it possesses the ability to attenuate their complete establishment [38]. Furthermore, the exogenous application of abscisic acid (ABA) significantly enhances the virulence of *C. acutatum*, indicating that the fungus can exploit the host’s ABA signaling pathway to suppress defenses [39]. Concurrently, the effector protein ChNLP1 from *C. higginsianum* achieves precise host infection through the chronological regulation of its expression [40]. These findings identify potential targets for advanced nano-enabled host-induced gene silencing (HIGS) and targeted intervention. Specifically, key virulence-associated regulators (e.g., CsCreA, CgCOM1) serve as ideal targets for nano-RNA interference (RNAi) platforms. Concurrently, the pathogen’s manipulation of host hormones and reliance on stage-specific effectors provide the mechanistic basis for designing stimuli-responsive smart nanomaterials (e.g., surface-modified or pH/sugar-triggered vehicles).

Furthermore, the discovery of mycoviruses provides a novel perspective for understanding virulence variation in *Colletotrichum*. These mycoviruses are predominantly composed of dsRNA genomes [41,42]. Recently, the first-time discovery of two entirely novel negative-sense single-stranded RNA viruses, CaNSRV1 and CaNSRV2, in a turfgrass-pathogenic *Colletotrichum* strain in Japan has expanded our understanding of the *Colletotrichum* virome diversity [43]. These viruses exert diverse effects on the phenotypes of their fungal hosts—for instance, CaPV1 can significantly increase the conidial production of *C. acutatum*, whereas CfCV1 reduces the growth rate and virulence of *C. fructicola* [42,44]. Consequently, dissecting these mycovirus strains opens up dual-track opportunities for nano-enabled disease management. On one hand, nanocarriers can be engineered to deliver specific dsRNA sequences that trigger gene silencing against virulence-enhancing viruses like CaPV1. On the other hand, bio-compatible nanoprotectants can shield naturally hypovirulent viral strains like CfCV1 from environmental degradation, thereby facilitating the development of stable and targeted biological control strategies.

## 3. Research Progress of Nanomaterials in the Control of Plant Anthracnose

Nanomaterials generally refer to functional materials with at least one dimension in the size range of 1–100 nm. Their unique quantum size effects, surface effects, and small-size effects grant them large specific surface areas, high surface reactivity, and finely tunable physicochemical properties that differ from those of conventional macro-scale materials [45]. In recent years, nanotechnology has emerged as a promising frontier approach for the green transformation of agriculture, offering a novel conceptual paradigm to address persistent bottlenecks in traditional plant disease management, such as low pesticide utilization efficiency, frequent emergence of pathogen resistance, and severe environmental pollution from pesticide residues [46]. In control systems against pathogenic fungi, the modes of action of nanomaterials can generally be categorized into two types: one comprises nanomaterials with intrinsic antimicrobial activity, which directly kill the pathogens through released ions, physical puncture, or electrostatic interactions (see Section 3.1); the other acts as delivery carriers to achieve targeted and controlled release and synergistic enhancement of chemical fungicides, biocontrol agents, and RNAi reagents, thereby significantly improving the bioavailability of the agents and reducing risks to non-target organisms (see Section 3.2) [47]. To date, nanotechnology has attracted substantial attention in research targeting the management of various critical plant fungal diseases, including anthracnose, presenting a diversified technological framework that encompasses multiple types of nanomaterials as well as nano-delivery systems loaded with chemical and biological agents [46,48]. However, it should be noted that the majority of current research findings are derived from laboratory (in vitro) or controlled-environment (e.g., greenhouse or detached organ) studies, with field-scale efficacy and practical agricultural applicability remaining to be fully verified.

### 3.1. Nanomaterials with Intrinsic Antimicrobial Activity Against Colletotrichum

Nanomaterials currently under laboratory and experimental evaluation for anthracnose management are primarily categorized into three major classes. Among these, metal and metal oxide nanoparticles represent the earliest studied and most extensively documented types. Concurrently, natural polymer nanomaterials and carbon-based nanomaterials are also exhibiting excellent prospects for industrialization due to their unique physicochemical properties and environmental friendliness [48,49].

#### 3.1.1. Metal and Metal Oxide Nanoparticles

Due to their unique physicochemical properties and broad-spectrum antimicrobial activities, metal and metal oxide nanoparticles have become a prominent research hotspot in green plant disease management. Among them, gold, silver, copper-based, and transition-metal oxide nanoparticles have demonstrated notable potential in experimental models of anthracnose (*Colletotrichum* spp.) management, with their mechanisms of action spanning direct fungicidal effects and the induction of plant systemic resistance (Table 1).

Gold nanoparticles (AuNPs) exhibit an innovative dual-action mode in the management of pepper anthracnose. Research indicates that 75 mg·L^−1^ of AuNPs can inhibit the mycelial growth of *C. gloeosporioides* by up to 61% and significantly reduce the spore germination rate to 25% in vitro. More critically, under controlled greenhouse conditions, AuNPs can effectively enhance the physiological and metabolic activities of pepper plants: on one hand, they markedly elevate physiological indicators such as total phenols, chlorophyll, free amino acids, and betaine in leaves while promoting the uptake and translocation of mineral nutrients such as phosphorus, nitrogen, zinc, and potassium (e.g., increasing leaf nitrogen and potassium contents by 152% and 113%, respectively); on the other hand, they systematically bolster the immune resilience of plants by activating the expression of defense-related genes and transcription factors [50]. Furthermore, “green synthesis” strategies utilizing biological resources have attracted significant attention for AuNP preparation, with “mycosynthesis”—which employs the anthracnose fungi themselves as bio-factories—showing considerable environmental and practical potential. For instance, an endophytic *Colletotrichum* sp. isolated from *Pelargonium graveolens* leaves [62], as well as *C. gloeosporioides* and *C. lindemuthianum* isolated from medicinal plants [63], can utilize their secreted extracellular proteins and secondary metabolites as reducing and capping agents to effectively reduce and stabilize gold ions into polydisperse spherical AuNPs with particle sizes ranging from 8 to 40 nm. This non-toxic, low-cost biosynthetic pathway offers new insights for the large-scale production of nanomaterials.

Owing to their broad-spectrum antimicrobial properties, silver nanoparticles (AgNPs) have become one of the most thoroughly investigated nanomaterials in anthracnose control research. In terms of in vivo control efficacy phenotypes, AgNPs exhibit disease-control potential comparable or superior to commercial fungicides. Foliar application of 50 ppm AgNPs can reduce the severity of pepper anthracnose by 90% in preliminary field trials, though long-term efficacy across continuous crop rotations remains to be systematically validated [64]. Laboratory studies have further confirmed that AgNPs significantly upregulate the expression of defense-related genes such as *CaPDF1.2* and *CaPR2* in pepper plants and enhance the activities of defense enzymes such as phenylalanine ammonia-lyase (PAL) and polyphenol oxidase (PPO), thereby reinforcing disease resistance by inducing host systemic resistance [65]. Research on common bean (*Phaseolus vulgaris*) in greenhouse experiments showed that 50 mg·L^−1^ of AgNPs reduced anthracnose severity by 90% and promoted plant growth, increasing shoot and root fresh weights by 17% and 90%, respectively [66]. Moreover, AgNPs have shown promising application prospects in forest disease management; studies have verified that they can effectively suppress the occurrence of anthracnose in *Catalpa bungei*, with a half-maximal effective concentration (EC_50_) for inhibiting mycelial growth of only 5 mg·L^−1^ in vitro [67]. Against different species within the genus *Colletotrichum*, AgNPs display a broad-spectrum inhibitory capacity. Studies have shown that AgNPs of 4–8 nm can effectively inhibit at least six pathogenic *Colletotrichum* species in vitro and in the field, including *C. gloeosporioides*, *C. acutatum*, *C. dematium*, *C. nigrum*, and *C. orbiculare* [64].

Although variations in efficacy exist across different particle sizes and concentrations of AgNPs, smaller-sized AgNPs (e.g., 5–24 nm) generally achieve an antimicrobial rate of nearly 90% at low concentrations (e.g., 56 µg·mL^−1^) in vitro; within this size range, the antimicrobial efficacy is primarily concentration-dependent rather than dictated by size variations [51]. Furthermore, by regulating glucose and gelatin concentrations as well as reaction temperatures, the particle size of AgNPs can be effectively controlled, with gelatin significantly enhancing colloidal stability through the interaction of its amide groups with the nanoparticle surface. In the exploration of green synthesis, beyond gelatin and glucose, researchers have successfully utilized natural resources such as cow milk [68], latex of *Calotropis procera* [69], and extracts of *Jatropha curcas* [70] to efficiently synthesize AgNPs, all of which exhibited excellent inhibitory effects against *C. gloeosporioides* or *C. musae*. Notably, the structural design and environmental activation of nanomaterials also profoundly influence their antimicrobial efficiency. For example, AgNPs capped with a bioactive bile acid salt (sodium deoxycholate, NaDC) exhibited significantly higher inhibitory efficiency against endophytic *C. gloeosporioides* upon photo-activation compared to dark treatments, without causing phytotoxicity to the host plant [71]. Additionally, the agricultural application of AgNPs must address clear dose-dependent boundaries and phytotoxic thresholds; while low concentrations effectively control pathogens, excessive silver accumulation poses risks of food chain migration and toxicity to beneficial soil microbiota. To circumvent these ecological risks, core–shell architectures and silica-silver composites (1–5 nm) have been designed [52,72]. Specifically, by slowly releasing Ag^+^ through a porous silica shell, Ag-SiO_2_ core–shell nanoparticles achieve fungicidal activity against *Colletotrichum* spore germination and *C. gloeosporioides* at extremely low concentrations (0.5–3 mg·L^−1^) through direct contact and ROS induction [52]. This approach not only reduces the required metallic dosage and mitigates bare AgNP oxidation, but also provides a viable strategy for addressing long-term soil accumulation concerns with minimal phytotoxic risks.

Copper-based nanomaterials demonstrate distinct advantages in the full-cycle management of plant anthracnose. For instance, Cu nanoparticles (CuNPs) synthesized by Ntasiou et al. [73] achieved a control efficacy of 71.7% against olive fruit anthracnose in a 20-year-old olive orchard, significantly outperforming conventional copper formulations. Mechanistically, these nanoparticles induce the accumulation of ROS within the pathogen, triggering oxidative stress that leads to mycelial membrane damage, hyphal malformation, and the leakage of intracellular contents. In vitro screening assays against mango anthracnose pathogens indicated that CuNPs-1, with a particle size of 5–10 nm, exerted the optimal inhibitory effect against four *Colletotrichum* species, with EC_50_ values ranging from 142.245 to 187.483 µg·mL^−1^, and preventive efficacy outperformed therapeutic efficacy [53]. The geometric morphology of nanoparticles also influences their antimicrobial activity. Octahedral cuprous oxide (Cu_2_O) nanocrystals synthesized via a one-pot method exhibited exceptional fungicidal efficiency against the pepper anthracnose pathogen (*C. capsici*) in vitro due to their unique exposed crystal facets and small-size effects [54]. Furthermore, an in vitro study on the tea anthracnose pathogen (*C. siamense*) revealed that treatment with 300 mg·L^−1^ nano-Cu(OH)_2_ for 2 days resulted in an inhibition rate of 99.16% [55]. In recent years, the advent of green synthesis technologies has further broadened the application value of copper-based nanomaterials. Joshi et al. [74] applied CuONPs biosynthesized using *Azadirachta indica* (neem) extracts to mango fruits, which reduced the post-harvest anthracnose incidence by 80% at a concentration of 100 ppm, while a 250 ppm treatment more effectively preserved fruit firmness (3.08 kg·cm^−2^) and vitamin C content (23.57 mg·(100 g)^−1^), thereby significantly extending the shelf life. Similarly, CuONPs synthesized using *Alpinia officinarum* extracts achieved a 74.69% in vitro inhibition rate against *Colletotrichum* mycelial growth at 1000 µg·mL^−1^, with a minimum inhibitory concentration (MIC) and minimum fungicidal concentration (MFC) of 125 µg·mL^−1^ and 500 µg·mL^−1^, respectively. These nanoparticles exerted their antifungal activity by disrupting hyphal morphology, triggering excessive ROS accumulation and lipid peroxidation, compromising cell membrane integrity, and consequently inducing the leakage of intracellular contents [56]. Despite these benefits, conventional copper accumulation in soils poses severe toxic risks to non-target soil microbiota and aquatic environments. To minimize metallic input, optimizing the morphology and dose dependency of Cu-based nanomaterials is critical, as exemplified by the aforementioned octahedral Cu_2_O nanocrystals that achieve a 90% inhibition rate against *C. capsici* at low dosages. Nevertheless, the potential phytotoxicity and ecological risks of copper nanoparticles at elevated concentrations (e.g., over 300 mg·L^−1^) must be strictly managed. Utilizing stabilizers (such as animal proteins or polymers) to regulate nanoparticle dispersion and release kinetics represents a crucial strategy to ensure superior protective efficacy on hosts like olive and pepper while minimizing copper residues in the agricultural environment [73].

Regarding studies on metal oxide nanoparticles for anthracnose control, zinc oxide nanoparticles (ZnONPs) and magnesium oxide nanoparticles (MgONPs) have been relatively widely researched. ZnONPs have garnered attention due to their low cost, Generally Recognized as Safe (GRAS) status, and broad-spectrum antimicrobial activity. In vitro assays involving a coffee anthracnose pathogen (*Colletotrichum* sp.), 15 mmol·L^−1^ (approximately 1200 ppm) of ZnONPs induced mycelial vacuolation and cytoplasmic liquefaction, achieving a 96% inhibition of mycelial growth [57]. In laboratory screening against mango anthracnose pathogens, ZnONPs exhibited EC_50_ values ranging from 269.370 to 568.816 µg·mL^−1^ against four common mango anthracnose fungi and effectively suppressed conidial germination and appressorium formation [53]. Additionally, functioning as nano-fertilizers, ZnONPs release Zn^2+^, an essential micronutrient for plant development, thereby exerting a synergistic effect in promoting healthy plant growth. Similarly, MgONPs display antifungal activity by inducing ROS generation and disrupting cell membrane integrity. Research indicates that in vitro assays and detached-leaf experiments show that 400 µg·mL^−1^ and 800 µg·mL^−1^ of MgONPs significantly inhibit the mycelial growth and spore germination of *C. gloeosporioides* in citrus, with early application at 400 µg·mL^−1^ providing superior preventive efficacy [58]. In screening against mango anthracnose pathogens, 800 µg·mL^−1^ of MgONPs even exhibited a lethal effect against *C. asianum* [53]. Similar to Cu-based materials, metal oxides such as ZnO, TiO_2_, and SiO_2_ also require a balanced assessment of their dose dependence and potential negative impacts, as their agricultural applications are inherently accompanied by a dose-dependent double-edged sword effect. While lower doses of ZnONPs can serve as essential micro-nutrients to promote growth, excessive application can cause significant phytotoxicity, alter plant physiological pathways, and disrupt beneficial soil microbiota. Furthermore, the persistent accumulation of Zn^2+^ and other metal ions in soil alters the soil microbial community structure and increases long-term environmental risks. Therefore, future applications of these metal oxide nanomaterials must strictly establish clear dose thresholds to leverage their nutritional and disease-control benefits without triggering ecological toxicity [52,57].

Beyond these, transition-metal composite oxide nanoparticles such as palladium nanoparticles (PdNPs) and cobalt/nickel ferrites have recently revealed novel antimicrobial attributes. Osonga et al. [59] discovered that PdNPs exert favorable in vitro inhibitory effects against both *Colletotrichum* and *Fusarium* species, with inhibition zone diameters reaching up to 12.2 mm. The underlying antimicrobial mechanism likely involves the binding of Pd^2+^ to DNA phosphate groups, alongside the inhibition of crucial metabolic enzymes such as succinate dehydrogenase and creatine kinase. Furthermore, platinum (Pt) and vanadium (V)-based nanomaterials have gradually demonstrated potential application value in anthracnose management. Spherical/elliptical platinum nanoparticles (PtNPs) of 10–50 nm green-synthesized using *Prunus × yedoensis* (Yoshino cherry) gum extract exhibited significant antifungal activity against *C. acutatum* in vitro [60]. Meanwhile, novel nano-sized oxovanadium (IV) complexes (18–24 nm) coordinated with Schiff base ligands achieved an in vitro inhibition rate of up to 76.0% (at 200 µg·mL^−1^) against the sugarcane red rot pathogen *C. falcatum*, while concurrently exhibiting broad-spectrum inhibitory activity against various Gram-positive and Gram-negative bacteria [61].

#### 3.1.2. Natural Polymer Nanomaterials

To reduce the residue risks associated with metal-based nanomaterials and to improve biocompatibility, natural polymers and their composite nanomaterials have emerged as a major research focus in the green control of anthracnose, owing to their biodegradability, environmental friendliness, and low toxicity to non-target organisms [75,76] (Table 2). Among them, chitosan and its derivatives are currently among the most intensively investigated and well-documented natural polymer substrates due to their inherent antibacterial activity, favorable film-forming properties, and cationic nature. Chitosan nanoparticles (ChNPs) prepared via an ultrasound-assisted top-down approach exhibit size-dependent antifungal activity. However, the antifungal efficacy of chitosan-based systems is highly dependent on key physicochemical properties, environmental parameters, pathogen types, and host systems [77]. Chemically, low-molecular-weight chitosan offers superior solubility and antimicrobial activity over high-molecular-weight counterparts, while a high degree of deacetylation exposes free amino groups crucial for electrostatic interactions and metal-ion binding. Environmentally, the primary amines (pKa ≈ 6.5) are only protonated into active cationic forms (-NH_3_^+^) under acidic conditions; at neutral or alkaline pH, the loss of charge causes precipitation and a decline in membrane-disrupting capabilities. Biologically, susceptibility varies across fungi due to differences in membrane lipids and cell wall thickness, while high concentrations of bulk chitosan can trigger host-specific phytotoxicity (e.g., restricted growth or tissue death). Thus, shifting from bulk chitosan to size-controlled submicron chitosan dispersions (SCD) or nano-hybrids is essential to optimize cellular delivery and bypass these limitations [77].

Specifically, when mini-sized into stable dispersions, at a concentration of 0.5%, uniform spherical particles with an average diameter of only 30 nm were obtained. These particles achieved a 97% in vitro inhibition rate against the mycelial growth of *C. gloeosporioides* on mango over 7 days, reducing the pathogen growth rate to 0.008 cm·h^−1^ (only 21% of the control group). This mechanism is primarily attributed to the enhanced cell membrane-penetrating ability resulting from their small size. ChNPs can adsorb onto the negatively charged fungal surfaces via electrostatic interactions, disrupting the integrity of the cell wall and membrane, causing the leakage of intracellular contents, and interfering with intracellular Ca^2+^ homeostasis, which ultimately leads to mycelial fragmentation, deformation, and cell death [75]. Concurrently, chitosan itself acts as a plant immune elicitor, significantly enhancing the activities of defense enzymes such as phenylalanine ammonia-lyase (PAL) and peroxidase (PO), thereby delaying disease onset and markedly reducing disease severity [77].

Building upon the application of natural polymers, the introduction of synthetic amphiphilic polymers for composite self-assembly can further optimize the physicochemical properties and interfacial behaviors of nanoparticles. For example, Yin et al. designed and synthesized a hydrophilic-lipophilic diblock polymer (HLDP), which self-assembled with chitosan (CS) through hydrophobic interactions to form a stable spherical nanocomposite (HLDP-CS). This process compressed the particle size of natural chitosan from 295 nm to 232 nm and elevated its surface Zeta potential to +72.83 mV. The extremely high positive charge density significantly enhanced the electrostatic adsorption between the nanoparticles and the negatively charged fungal mycelia, directly destroying the integrity of the anthracnose cell wall and membrane and effectively inhibiting spore germination. Furthermore, this composite system significantly reduced the contact angle of the pesticide solution on hydrophobic leaf surfaces, improving its deposition and retention capacity on mango leaves, thus providing empirical support for the modification of natural nanomaterials using synthesized polymers [78].

To further enhance broad-spectrum fungicidal efficacy and application stability, researchers have developed various chitosan–metal and chitosan–metal oxide composite systems. Chitosan–copper nanocomposites (CHT-CuNPs) synthesized via the ionotropic gelation method exhibit an average particle size of approximately 153.8 nm and a surface charge of +22.2 mV. They display an in vitro minimum inhibitory concentration (MIC) of 400 µg·mL^−1^ against citrus *C. gloeosporioides* and show significant antagonistic activity against various soil-borne and foliar pathogens, such as *Fusarium ciceri* and *Sclerotium rolfsii*, with efficacy significantly superior to that of chitosan or copper sulfate alone [76]. Additionally, chitosan–silver nanocomposites (CS-AgNPs), prepared using low-MW chitosan as both a reducing and stabilizing agent under alkaline conditions at 90 ± 1 °C, enable precise control of silver nanoparticle size within the 4–15 nm range. The in vitro inhibitory effect of this composite material on the spore germination of *C. truncatum* (pepper anthracnose) far exceeds that of chitosan alone. Even at a low concentration of 0.0625%, it significantly promotes pepper seed germination and seedling growth, functioning dually in disease prevention and growth promotion [79]. Besides chitosan, other natural polymers such as alginate have also attracted systematic research attention. For instance, alginate microbeads loaded with titanium nanoparticles (TiNPs) and chitosan nanoparticles have shown efficacy in controlling maize anthracnose stalk rot in greenhouse pot experiments, with overall efficacy surpassing that of traditional chemical fungicides, demonstrating their potential for large-scale application as eco-friendly biofungicides [6].

#### 3.1.3. Carbon-Based Nanomaterials

Carbon-based nanomaterials (such as carbon quantum dots, graphene, and graphene oxide) have demonstrated broad application prospects in the green control of plant fungal diseases in recent years, owing to their unique two-dimensional layered structures, ultra-high specific surface areas, excellent physicochemical stability, and biocompatibility.

In postharvest disease management of fruits and vegetables, carbon-based materials exhibit notable direct antifungal efficacy. Carbon quantum dots (DE-CQDs) green-synthesized from the agricultural byproducts of *Dictyophora echinovolvata* demonstrated remarkable antifungal efficacy against *C. gloeosporioides*. DE-CQDs could disrupt the mycelial structure of the pathogen, causing the hyphae to become severely shriveled, rough, and flattened, thereby drastically weakening its infection capability. When incorporated as a functional filler into a xanthan gum/hydroxypropyl methylcellulose (XG/HPMC) matrix film, the resulting XHC composite film showed a 30% increase in UV-blocking capacity alongside appropriate moisture permeability. Postharvest preservation experiments on mangoes indicated that the XHC coating treatment significantly reduced the 7-day decay index of mangoes from 0.35 to 0.21, effectively extending shelf life and maintaining nutritional quality [80].

Aside from directly targeting pathogens, graphene oxide (GO) can indirectly influence disease occurrence by modulating the rhizosphere micro-ecology. To manage peach anthracnose, researchers co-applied GO and *Bacillus velezensis* to the rhizosphere of infected trees. Evaluation of actual control efficacy revealed that the “microbe-GO” composite treatment yielded positive soil-conditioning effects: it was reported to mitigate the soil pH imbalances associated with the disease, reduce soil calcium and zinc concentrations, and promote soil organic carbon. Macroscopic outcomes indicated that this combined strategy helped stabilize the rhizosphere ecology of diseased peach trees, decreasing orchard anthracnose severity [81]. However, these rhizosphere data remain highly localized, and further replication is required to confirm whether this “soil health remediation” effect can be generalized across diverse soil types and different orchard systems without causing negative long-term soil carbon accumulation issues.

Furthermore, based on the concept of green circular agriculture, nano-biochar prepared from agricultural wastes has further expanded the source of carbon-based antimicrobial materials. One in vitro study showed that colloidal/nano-biochar prepared from corn cobs and *Gliricidia sepium* wood effectively inhibited the mycelial growth of *C. musae* within a concentration range of 0.4–20 g·L^−1^, significantly reducing mycelial density. The addition of zeolite further enhanced its antifungal efficacy, providing new insights into utilizing agricultural wastes to develop sustainable materials for anthracnose control [82].

### 3.2. Advances in the Application of Nano-Delivery Systems for Anthracnose Control

Nanomaterials serve not only as direct antimicrobial agents but also as promising carriers for constructing intelligent, multifunctional pesticide delivery systems. Depending on the properties of the encapsulated agents, nanocarriers exhibit significant advantages in enhancing efficacy, providing stabilization and protection, and facilitating transbarrier delivery.

#### 3.2.1. Targeted Release and Interface Optimization of Nano-Chemical Fungicides

In the field of chemical pesticide delivery, nanocarriers significantly enhance the comprehensive control efficacy of traditional fungicides by protecting active ingredients from photolysis, enabling targeted delivery, and improving foliar interfacial properties (Table 3). Studies have demonstrated that formulating traditional fungicides (e.g., azoxystrobin, tebuconazole, and prochloraz) into nano-formulations can substantially improve their control efficacy through particle size regulation, surface modification, and dispersion state optimization. For instance, drug-loaded nanospheres formed by encapsulating azoxystrobin with biodegradable materials like polylactic acid (PLA) can significantly optimize their biological activity via particle size control; notably, small-sized nanospheres (130.9 nm) increased the relative toxicity against *C. higginsianum* by more than 10-fold [83], representing a promising laboratory-scale prototype evaluated through in vitro toxicity assays. Targeting strawberry anthracnose, chitosan/O-carboxymethyl chitosan/tebuconazole nanoparticles (CS/O-CMCS/TBA NPs) prepared by Wu et al. [84] leveraged the rapid release and efficient transmembrane transport capabilities associated with their small particle size to induce excessive ROS accumulation and trigger apoptosis in fungi. This enhanced the disease resistance activity by 3.13 times compared to traditional suspension concentrates, providing a verified biocompatible prototype ready for further field trials.

Inorganic porous materials, such as metal–organic frameworks (MOFs) and mesoporous silica nanoparticles (MSNs), exhibit unique advantages in the controlled release and targeted delivery of agrochemicals due to their high specific surface areas and ease of modification. However, evaluations of most of these sophisticated systems currently remain restricted to the in vitro or controlled greenhouse stages due to their complex synthesis and the reliance on simulated or indoor microenvironments. For example, Pro@ZIF-8, formed by loading prochloraz into the pH-responsive zeolitic imidazolate framework-8 (ZIF-8), can release 89.8% of its active ingredients within 24 h under acidic conditions (pH 5.0). Its EC_50_ against *C. fructicola* was reduced to 0.0150 mg·L^−1^, significantly lower than that of free prochloraz (0.0429 mg·L^−1^), which successfully delayed lesion expansion on strawberry leaves and fruits in indoor and postharvest storage quality assessment [85]. MSNs dual-functionalized with carboxymethyl chitosan (CMC) and a mitochondria-targeting moiety (TPP) enabled precise targeted delivery of azoxystrobin (a mitochondrial respiratory chain inhibitor) with a drug-loading capacity of 32%. Its antifungal efficacy at low dosages remained significantly superior to conventional commercial formulations in in vitro bioassays [86]. Furthermore, a nano-delivery system based on mesoporous silica/carboxymethyl β-glucan loaded with chlorothalonil not only exhibited superior biological activity against various pathogens, including *C. truncatum*, compared to commercial formulations, but also reduced toxicity to non-target organisms (earthworms and zebrafish) by 2.25 and 2.66 times, respectively, achieving a balance between efficacy enhancement and ecological safety [87]. While this system balances efficacy enhancement and ecological safety, it still requires validation under complex, open-field agricultural environments.

In field disease control, constructing multicomponent nanocomposite systems with interfacial anti-washout and microenvironment-responsive characteristics is a core strategy for mitigating off-target pesticide losses, facilitating the transition from laboratory-scale conceptualization to practical field validation. For instance, modifying the surface of reduced graphene oxide (rGO) nanosheets with broad-spectrum antimicrobial Cu_2−x_Se nanocrystals, compounding with captan, and introducing a chitosan coating and cationic polymer modification increased the pesticide’s binding rate on plant leaves by 30% and reduced off-target soil leaching by 45%, achieving a 50% growth inhibition rate against *C. capsici* [5], which demonstrates great potential for rain-washout resistance in field applications. An amphiphilic polypeptide-brush nanocarrier (CXP-NH_3_) constructed from cellulose and cationic antimicrobial peptides, when loaded with trifloxystrobin, similarly utilized its robust foliar adhesion to dramatically increase the drug retention rate from 18% to 67% after simulated heavy rainfall washout [88]. Crucially, some advanced formulations have successfully advanced to the stage of authentic field validation. The DIF/CuS@Cu-MOF system, synthesized via an in situ vulcanization method, not only significantly prolonged the duration of efficacy but also enhanced drug retention by 36.5-fold and 9.4-fold on structurally distinct cucumber (hydrophilic) and peanut (hydrophobic) leaves, respectively, culminating in a verified 43.9% improvement in control efficacy against *C. gloeosporioides* under real field conditions [89].

In summary, current nano-chemical pesticide research exhibits a distinct structural bifurcation. Highly precise, stimulus-responsive carriers utilizing multi-functionalized MSNs or advanced MOFs offer enhanced targeted delivery but remain largely confined to the in vitro or controlled greenhouse stages due to structural complexity, high production costs, and a lack of open-field data. Conversely, systems optimizing the foliar interface and anti-washout capabilities via eco-friendly polymeric coatings or hybrid matrices have successfully transitioned into the field-validation or practical application phase. Whether relying on particle size regulation, the smart responsiveness of porous carriers, or anti-washout modifications based on the foliar microenvironment, nano-chemical pesticide systems can effectively overcome the bottlenecks of traditional agents, such as susceptibility to off-target loss and low utilization rates. This provides crucial technical support for achieving the “reduction of application and enhancement of efficacy” of chemical pesticides and promoting green, sustainable disease control.

#### 3.2.2. Stable Delivery and Synergistic Enhancement of Nano-Biopesticides

Regarding the delivery of biological control agents and biogenic active substances, nanomaterials can serve as protective shells and synergists for biocontrol microorganisms, active essential oils, and antimicrobial peptides, addressing the challenges of high volatility, rapid degradation, and poor colonization in the environment. For instance, a thermostable chitinase mutant (I168L) extracted from *Trichoderma asperellum* was encapsulated in alginate and complexed with ZnONPs. The resulting complex retained 95% of its activity after 30 days of storage. This system demonstrated favorable control efficacy against *Colletotrichum* species on pitaya; the chitinase initially degraded the fungal cell walls, creating favorable conditions for the penetration of ZnONPs, thereby achieving an “enzyme-nano” synergistic effect [90].

For the stable delivery of plant-derived essential oils, a composite coating prepared using 0.8% chitosan loaded with 1% thyme essential oil nanoemulsion completely inhibited the spore germination of *C. gloeosporioides* in in vitro assays and reduced the disease incidence on tomatoes by 50% in in vivo experiments [91]. In the postharvest control of anthracnose in avocado (cv. Hass), a nano-biocomposite coating formulated by encapsulating *Schinus molle* essential oil within chitosan exhibited dual prophylactic and therapeutic activities. Ten days after artificial inoculation with *C. gloeosporioides*, the treated fruits showed no significant internal decay. Concurrently, acting as a semi-permeable modified-atmosphere film, this coating restricted fruit moisture loss to approximately 1% and decelerated firmness loss to 80% (compared to 96% in the control group), thus significantly maintaining postharvest fruit quality [92]. In the delivery of biological antimicrobial peptides, natural minerals demonstrate unique structural advantages. Montmorillonite (MMT), a natural layered silicate mineral, was utilized to load the cationic peptide aptamer SNP-16 via electrostatic interactions. This not only increased the peptide loading rate to 80% but also significantly enhanced the adhesion of the peptide to *Stylosanthes* leaf surfaces via hydrophobic interactions. Field trials indicated that a mere 0.1 mg dose of the MMT/SNP complex achieved a control efficacy against *Stylosanthes* anthracnose comparable to that of 1% commercial chemical fungicide carbendazim [93].

However, the practical application of nano-biopesticides faces critical ecological risks and technical bottlenecks. Although nano-encapsulation extends the environmental persistence of biocontrol agents [90,93], their small particle size increases the risk of non-target distribution via drift or runoff, potentially harming aquatic and terrestrial ecosystems. Furthermore, these nano-systems can disrupt the homeostasis of beneficial soil and phyllosphere microbial communities. Technically, maintaining formulation stability remains challenging under thermal stress and extended storage [90].

#### 3.2.3. Efficient Delivery and Target Gene Silencing of Nano-RNAi Systems

Currently, leveraging nanomaterials as delivery vehicles for RNAi reagents has emerged as a research focus in agricultural disease management. RNAi technology achieves disease control by target-silencing key genes in pathogens (such as those involved in spore germination, appressorium formation, cell wall synthesis, and metabolic regulation), offering high specificity and environmental compatibility [94,95]. However, naked dsRNA is highly susceptible to enzymatic degradation by nucleases in the environment. It also faces significant challenges in penetrating plant cell walls and pathogen cellular barriers, alongside exhibiting vast differences in delivery efficiency across diverse species. These bottlenecks severely restrict the field application of RNAi technology [96]. To address these challenges, RNA biopesticides based on nano-delivery systems represent a core strategy, as they can substantially enhance the environmental stability and delivery efficiency of dsRNA [97,98,99]. Although studies have shown that several pathogens, including *C. gloeosporioides* and *Phytophthora infestans*, cannot directly take up naked dsRNA from the environment [100], HIGS research has demonstrated that transgenic tomato and pepper plants stably expressing RNAi constructs targeting the *CgCOM1* gene of *C. gloeosporioides* can effectively inhibit conidial germination and appressorium formation [28]. This evidence indicates that *C. gloeosporioides* possesses a latent mechanism for internalizing exogenous (plant-derived) dsRNA and triggering RNAi, thereby providing a crucial theoretical foundation for the exogenous application of dsRNA via nanocarriers.

However, in developing non-transgenic Spray-Induced Gene Silencing (SIGS) for *Colletotrichum*, a major biological and physical barrier is the cell wall of the mature appressorium. Unlike more permissive fungi with thin hyphal walls (e.g., *Botrytis cinerea*), the mature *Colletotrichum* appressorium is covered by a heavily melanized, highly cross-linked, rigid chitin-glucan cell wall [76]. This dense physical structure acts as an extremely restrictive barrier that limits the uptake of exogenous naked dsRNA or large nanocarrier complexes [101]. Unless the nanocarriers are specifically designed to be extremely small or are functionalized to transiently compromise wall integrity (e.g., co-delivery with chitin-softening agents), the physical exclusion by the melanized appressorium wall may severely impede RNAi efficiency in the pre-penetration phase.

Accumulating evidence indicates that complexing dsRNA with nanomaterials—such as chitosan, star polycation (SPc), or layered double hydroxides (LDHs)—not only effectively protects dsRNA from degradation by environmental nucleases but also facilitates its penetration through fungal cell walls into the intracellular space, thereby achieving highly efficient gene silencing [101]. Taking the SPc nanocarrier as an example, its cellular uptake and intracellular trafficking mechanisms are characterized by two parallel pathways: on one hand, it significantly enhances the uptake efficiency of dsRNA by target cells through activating clathrin-mediated endocytosis; on the other hand, it exploits the “proton sponge” effect to facilitate endosomal escape of dsRNA into the cytoplasm, thereby triggering the RNAi pathway [102,103]. Although specific cases of nano-RNA biopesticides targeting *Colletotrichum* species have not yet been reported, the aforementioned nano-delivery strategies have demonstrated remarkable efficacy in controlling various fungal diseases caused by pathogens such as *Magnaporthe oryzae* and *B. cinerea*. These findings provide indispensable theoretical support and technical insights for the future development of highly efficient and stable dsRNA nano-pesticides specifically engineered against pathogenic targets in *Colletotrichum* [101].

### 3.3. Comparative Synthesis of Nanomaterial Classes

While previous discussions outline the broad potential of individual nanomaterials, a critical cross-cutting synthesis is essential to objectively weigh their respective advantages against inherent practical limitations (Table 4). Furthermore, an honest appraisal must acknowledge that the current body of evidence for these materials predominantly relies on in vitro laboratory evaluations, with a distinct scarcity of comprehensive greenhouse and large-scale open-field validations [61,87].

Among the intrinsically active materials, metal and metal oxide nanoparticles consistently demonstrate acute fungicidal efficacy, achieving substantial pathogen inhibition at relatively low concentrations via rapid ROS generation and ion release [51,53]. Their primary appeal lies in this robust, broad-spectrum biocidal activity. However, these benefits are strictly counterbalanced by severe environmental liabilities, including high environmental persistence, heavy metal bioaccumulation in soils, and pronounced phytotoxicity risks toward non-target crops and beneficial soil microbiomes [61,87]. Elevated local accumulations can inhibit beneficial soil nitrifying bacteria and disrupt plant-microbe symbioses, while agricultural runoff introduces acute toxicity risks to non-target aquatic organisms [61,87]. Consequently, their practical viability is heavily restricted, necessitating strictly controlled, low-dose applications or integration into protective core–shell architectures (such as Ag-SiO_2_ composites) to mitigate ecological toxicity and minimize metallic input [52,53].

Natural polymer-based nanomaterials, such as chitosan and alginate, offer an alternative profile characterized by biocompatibility, biodegradability, and dual functionality, serving simultaneously as physical antimicrobial barriers and elicitors of host plant immunity [75,78]. Although they present minimal environmental risks, a notable disadvantage is their comparatively moderate direct fungicidal potency. Additionally, their structural and functional stability is highly vulnerable to environmental fluctuations, such as shifts in pH; for instance, the primary amines of chitosan require acidic conditions for adequate protonation and activation [75]. Overcoming these limitations necessitates shifting from bulk polymers to size-controlled submicron dispersions or synthesizing composites with amphiphilic polymers (e.g., HLDP-CS) to enhance positive charge density, membrane-penetrating ability, and foliar deposition [78]. Thanks to superior film-forming characteristics, these materials currently find their most practical utility in post-harvest fruit preservation through edible coatings [75,78].

Carbon-based nanomaterials bring unique structural attributes, including tunable surface chemistry and high specific surface areas, rendering them promising for modulating the rhizosphere microenvironment and physically blocking pathogen infection. Materials like graphene oxide have demonstrated potential in conditioning diseased soils by mitigating pH imbalances and promoting organic carbon [81]. Despite these physicochemical advantages, practical translation is severely constrained by high manufacturing costs, an absence of rigorous field-scale safety validations, and highly uncertain long-term environmental fates within agricultural food chains. At present, application is largely restricted to functional adjuvants or specialized composite fillers within controlled settings [81].

Distinct from intrinsically active materials, advanced nano-delivery systems represent a sophisticated carrier strategy designed to encapsulate and protect active chemical or biological agents, thereby bypassing traditional formulation pitfalls like photodegradation, leaching, and poor rain-fastness [84,88]. Their standout feature is the capacity for targeted, stimuli-responsive release triggered by microenvironmental cues like pH or enzymatic activity, which markedly enhances overall utilization efficiency [85,86,87]. However, highly precise inorganic porous carriers (such as MOFs and MSNs) currently face significant scale-up cost barriers and critically lack extensive open-field efficacy data [85,89]. While delivery systems utilizing eco-friendly polymeric coatings or hybrid matrices have shown progress in optimizing foliar adhesion, hurdles such as complex synthesis procedures and long-term storage stability must be rigorously addressed before widespread agricultural deployment can be realized [88,89].

## 4. Molecular Mechanisms of Nanomaterials Against Colletotrichum

**Figure 2:**
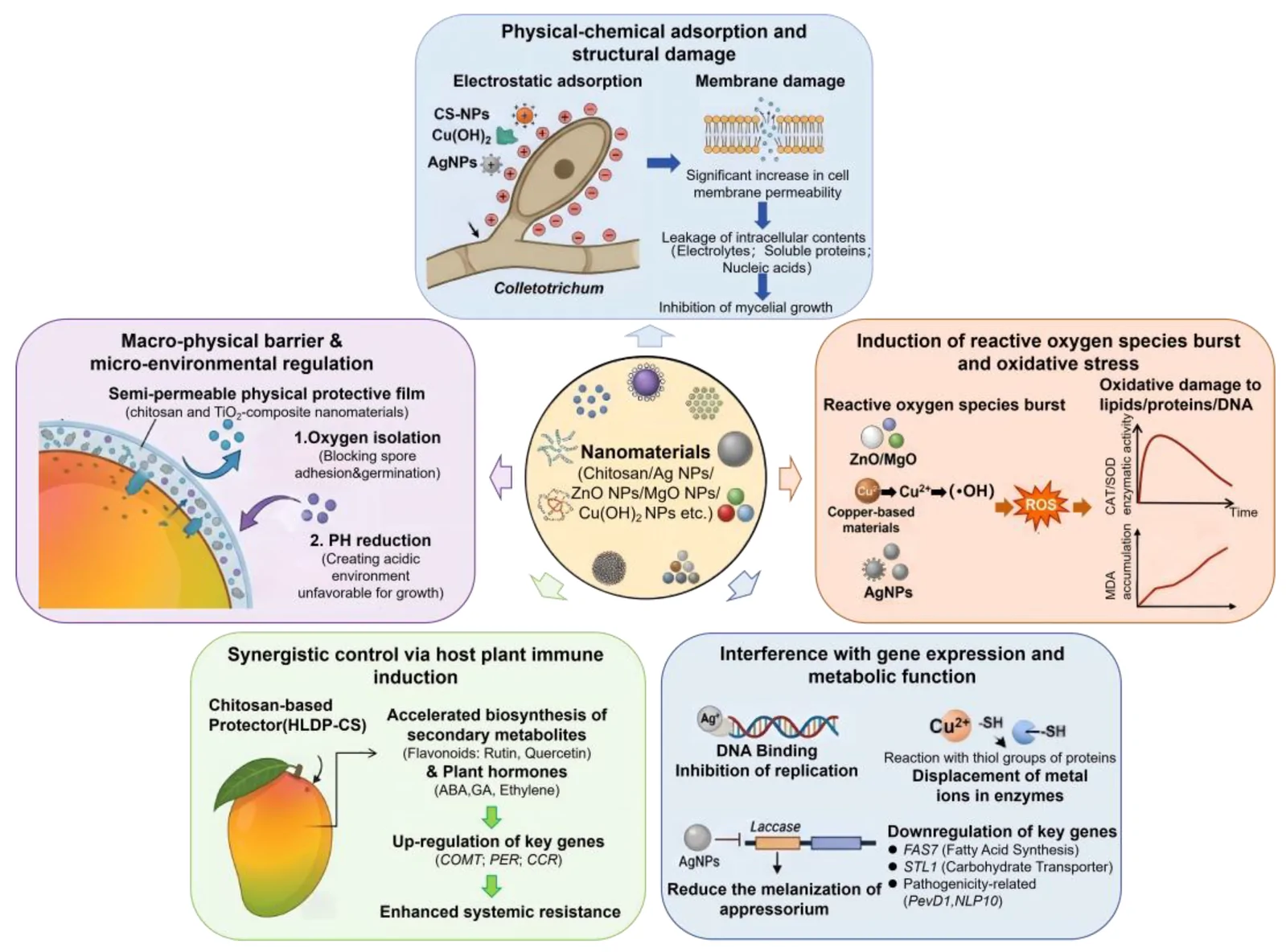
Molecular mechanisms of nanomaterial-mediated control of anthracnose.

The mechanisms of action of nanomaterials against *Colletotrichum* are multifaceted, primarily inhibiting its growth through various pathways such as direct physicochemical damage and the induction of intracellular oxidative stress within the fungus. These mechanisms often occur synergistically, as illustrated in Figure 2.

Nanomaterials can inhibit pathogen growth by directly damaging cell structures and disrupting physical barriers, which constitutes the most direct and rapid mode of their antifungal action. Because the surfaces of *Colletotrichum* cell walls and membranes are typically negatively charged, while the surfaces of nanomaterials like chitosan nanoparticles (CSNPs) and Cu(OH)_2_ nanoparticles are positively charged, this strong electrostatic interaction enables the nanomaterials to tightly adsorb onto the surfaces of hyphae and spores [55,104]. Direct biophysical and microscopic evidence reveals that this electrostatic affinity drives an immediate, irreversible disruption of the physical barriers; for instance, under nano-Cu(OH)_2_ exposure, electron microscopy and live-dead staining clearly visualize the rapid shrinkage, collapse, and fragmentation of fungal structures. This structural damage compromises the integrity of the cell membrane, thereby increasing its permeability [55]. The loss of the cell membrane’s barrier function results in a massive leakage of intracellular substances, including electrolytes, soluble proteins, and nucleic acids, ultimately leading to the arrest of hyphal growth and subsequent cell death [105,106].

As nanomaterials penetrate these physical barriers and invade the fungal cells, they disrupt the intracellular redox balance, triggering a severe oxidative stress response. Nanoparticles such as ZnO and MgO can spontaneously generate ROS in aqueous environments, while copper ions released from copper-based nanomaterials can catalyze the production of highly reactive hydroxyl radicals (•OHs) via the Fenton reaction [107]. Similarly, AgNPs have been confirmed by fluorescent probes to trigger a severe ROS burst [55,106]. This primary oxidative stress can be tracked in real time using fluorometric markers (such as DCFH-DA), which demonstrate a synchronized burst of ROS that mounts a broad attack on lipids, proteins, and DNA. This excessive accumulation causes severe structural damage to the spores of *C. gloeosporioides*, such as vacuole expansion, swelling, and melanization. To cope with this intense oxidative stress, the fungus urgently activates its endogenous antioxidant defense system, which may result in a transient initial increase in the activities of enzymes like SOD and catalase (CAT). However, under continuous nanomaterial-induced stress, the activities of SOD, CAT, and other enzymes are significantly inhibited, accompanied by a substantial accumulation of the lipid peroxidation product MDA, further confirming the occurrence of oxidative damage [55,106].

Concurrently with inducing oxidative stress, nanomaterials and their released ions directly interfere with the core physiological and metabolic functions of the fungus. Intracellular silver ions (Ag^+^) can bind to DNA, depriving it of its replication ability [51], while severe stress from copper-based nanomaterials has been shown to induce direct genomic degradation, manifested as a significant depletion of total intracellular DNA concentration [55]. Copper ions (Cu^2+^) can react with the sulfhydryl (-SH) groups of proteins or competitively displace essential metal ions in key enzymes, leading to the inactivation of various enzymes (e.g., ATPase), fundamentally blocking cellular energy metabolism and substance synthesis [55,106]. Beyond inhibiting basic physiological metabolism, nanomaterials can also attenuate the virulence of *Colletotrichum*. Studies have shown that AgNPs treatment can inhibit the expression of the laccase gene associated with melanin synthesis, thereby reducing the melanization of appressoria, a crucial step for the successful infection of host plants by *Colletotrichum* [66]. Transcriptomic sequencing analysis reveals that nanomaterials can broadly downregulate fungal gene expression, including key pathogenic pathways involving fatty acid synthesis-related genes (*FAS1*), sugar transporter family members (*STL1*), and pathogenicity-related genes (such as *PevD1* and *NLP10*), indicating their ability to reduce the pathogen’s virulence at the source [78].

Notably, in addition to direct antifungal activity, nanomaterials can achieve synergistic disease control by inducing host plant immune responses. Research indicates that treating mango fruits with a chitosan-based nano-protectant (HLDP-CS) can significantly amplify the plant’s endogenous immune response by accelerating the biosynthesis of secondary metabolites (such as flavonoids like rutin and quercetin) and phytohormones (such as abscisic acid, gibberellic acid and ethylene). Combined transcriptomic and metabolomic analyses have revealed that key genes (e.g., *COMT*, *PER*, *CCR*) and related metabolites in the phenylpropanoid and flavonoid biosynthesis pathways are significantly upregulated post-treatment, thereby enhancing the systemic disease resistance of the fruit [78]. Critically, the strength of evidence supporting these mechanisms varies. Direct antimicrobial effects—such as electrostatic membrane disruption, cellular leakage, and ROS-triggered oxidative stress—are firmly backed by robust, reproducible in vitro physicochemical data. In contrast, high-order regulatory mechanisms, including host systemic immunity activation and RNAi silencing, exhibit higher variability and are often contingent upon specific host–pathogen systems.

Furthermore, when utilized as coatings or films in postharvest fruit and vegetable preservation, nanomaterials exert significant macroscopic physical barrier and microenvironmental regulatory effects. Taking film-forming nanomaterials like chitosan and nano-TiO_2_ composites as examples, they can form a semi-permeable physical protective film on the surface of fruits or plants [107,108]. This film not only effectively blocks oxygen and directly eliminates the spatial conditions for spore adhesion and germination [108], but also lowers the pH of the fruit surface, creating an acidic microenvironment unfavorable for the growth of pathogens such as *C. gloeosporioides* [107].

## 5. Challenges and Limitations of Nanomaterials in Anthracnose Management

Although nanomaterials exhibit remarkable antimicrobial efficacy in vitro, their translation from laboratory research to practical field applications is hindered by multiple constraints:

### 5.1. Inadequate Biosafety and Ecotoxicity Risk Assessment

This constitutes the primary challenge currently confronting nanopesticides. While extensive research has focused on their antimicrobial activity, systematic evaluations of the long-term ecotoxicological effects of nanomaterials remain scarce. Specifically, the persistence, structural transformations, and biological behaviors of inorganic nanoparticles (such as AgNPs and metal-oxide NPs) within soil ecosystems require rigorous scrutiny [61,87]. Once introduced into the soil via run-off or foliar washing, these particles undergo complex physicochemical interactions—including dissolution, oxidation, aggregation, and surface adsorption onto organic matter—that directly govern their biological availability and prolonged toxicological profiles [87]. At the soil microbial level, high local accumulations of copper- or silver-based nanoparticles have been reported to inhibit beneficial soil nitrifying bacteria and nitrogen-fixing microflora, thereby disrupting essential soil nutrient cycling processes. Furthermore, these inorganic particles interfere with mutualistic plant-microbe symbioses by impairing mycorrhizal fungal colonization and altering the structural balance of beneficial rhizosphere microbial communities [61,79]. In terrestrial fauna, nanoparticles accumulate inside soil macro-organisms like earthworms (*Eisenia fetida*), entering via dermal absorption or direct ingestion [87]. This internal accumulation triggers ROS overproduction, lipid peroxidation, and cellular organelle damage, ultimately impairing growth, reproductive capacity, and soil bioturbation activity [87]. Beyond the soil matrix, agricultural runoff transports free nanoparticles and their transformation products into aquatic ecosystems, posing acute toxicity risks to non-target aquatic organisms (e.g., *Daphnia magna* and zebrafish *Danio rerio*) through gill surface coating, membrane rupture, and systemic oxidative stress [87]. Crucially, their potential transfer, tissue translocation, and biomagnification through the agricultural food chain remain almost entirely unexplored, posing unquantified risks to higher trophic levels and human food safety [61,87]. Therefore, establishing standardized, long-term multi-trophic ecotoxicity screening protocols remains imperative to ensure the ecological safety of nano-enabled plant protection strategies.

### 5.2. Insufficient Stability and Persistence in Complex Field Environments

The stability and persistence of nanosystems face significant challenges in complex natural environments. The ideal efficacies observed at the laboratory scale are difficult to fully replicate under highly variable field conditions. Environmental abiotic factors can dramatically alter the physical state and chemical behavior of applied nanomaterials: solar UV radiation can trigger photo-reduction or rapid oxidation of metal nanoparticles, leading to premature inactivation; frequent rainfall events wash away hydrophilic carriers; while fluctuations in soil/foliar pH, clay minerals, organic matter, and leaf epicuticular waxes can trigger rapid particle agglomeration or flocculation [108]. Consequently, their designed controlled-release kinetics can fail to match the actual epidemiology of disease development [73].

### 5.3. Pathogen Susceptibility Variations and Potential Resistance Risks

The inhibitory efficacy of a single nanomaterial can vary significantly across different anthracnose-causing species (and even among different strains of the same species). For instance, the half-maximal effective concentration EC_50_ values of ZnO and MgO nanoparticles against *C. fructicola* are substantially higher than those for other strains, indicating inherent variations in susceptibility [53]. Furthermore, fungal pathogens are highly adaptive; under chronic, sub-lethal exposure to metallic or natural polymer nanoparticles, pathogens can select for stress-tolerance mutations. These can include upregulation of multi-drug resistance (MDR) efflux pumps, alteration of cell wall composition (e.g., thick chitin deposition to block particle binding), enhanced extracellular biofilm formation, or hyper-activation of intracellular ROS-scavenging enzyme systems (SOD/CAT), which could lead to resistance over time.

### 5.4. The Dichotomy Between Scalable Production Costs and Practical Field Formulation Compatibility

The economics and industrial scalability of agricultural nanomaterials present a major practical bottleneck. While simple green-synthesized metal nanoparticles are relatively cheap to produce at a laboratory scale, highly engineered nanocarriers—such as MSNs, MOFs, and customized polymer-micelles—require complex multi-step chemical syntheses, organic solvents, and expensive instrumentation. Comparing these production costs against low-margin agricultural commodity values reveals a significant economic disparity. Furthermore, from a formulation perspective, serious compatibility gaps remain: many nano-suspensions exhibit poor long-term storage stability, suffering from Ostwald ripening or irreversible sediment aggregation under varying temperatures. Beyond storage-related challenges, aligning these novel formulations with conventional agricultural machinery and existing spraying equipment continues to be a significant challenge [109]. Ultimately, technology scalability and production costs are the primary factors that largely determine the feasibility of the practical use of nanomaterials, and addressing these economic and technical barriers is strictly essential for their successful commercial implementation.

### 5.5. Lack of Standardized Regulatory Frameworks and Registration Protocols

Currently, the regulatory pathway for nanomaterial commercialization is hindered by profound policy uncertainties. Standard regulatory protocols designed for conventional pesticides (such as testing active ingredient mass percentages) are not suited for assessing nanomaterials, where biological activity is dictated by physical particle size, surface charge (Zeta potential), and morphological properties. There are significant regulatory gaps and lack of standardized evaluation protocols for nanopesticides, nano-enabled biopesticides, and RNAi nanocarriers worldwide. Key issues—such as how to define the “nanopesticide” category, how to test long-term food-residue limits of synthetic nanocarriers, and how to govern the field release of genetic-silencing dsRNA structures—remain undefined by international regulatory bodies, posing a massive market barrier for agrotechnology developers. Therefore, the establishment of clear registration protocols and robust regulatory control mechanisms is urgently needed, as these elements play a decisive role in determining the practical feasibility and large-scale commercialization of nano-enabled agricultural products.

## 6. Future Perspectives

Despite various challenges, the application of nanotechnology in the prevention and control of plant diseases, such as anthracnose, is advancing toward more precise, intelligent, and green directions. Crucially, bridging the gap between innovative laboratory prototypes and commercial agricultural products requires a concerted focus on the practical dimensions of technology translation. Future advancements must be anchored in three foundational pillars: establishing robust pathways for regulatory approval, conducting comprehensive environmental risk assessments across diverse agroecosystems, and executing multi-regional, large-scale field validations. Addressing these practical bottlenecks is imperative to ensure that nano-enabled management strategies demonstrate consistent efficacy, economic viability, and ecological safety in real-world farming environments.

### 6.1. Early Pathogen Detection and Early Warning Based on Nanobiosensors

*Colletotrichum* species exhibit typical latent infection characteristics; therefore, highly sensitive detection prior to the appearance of visible symptoms is the foundation for timely and early intervention. Nanomaterials, such as quantum dots, functionalized dendrimers, and gold nanoshells, exhibit excellent optical and electrochemical properties, offering new possibilities for the development of highly specific nanobiosensors. Coupled with cascade nucleic acid signal amplification strategies (such as strand displacement amplification and hybridization chain reaction), these sensors have the theoretical potential to specifically target and recognize ultra-low concentrations of pathogen nucleic acids, secreted effector proteins, or early mycotoxins [109]. Nevertheless, notable technical hurdles remain regarding how such systems maintain target selectivity and stability in complex agricultural environments influenced by plant residues and environmental fluctuations. To address these limitations, the exploration of smartphone-assisted multimodal biosensing platforms—integrating electrochemical, colorimetric, and photothermal detection modes—provides a potential pathway for cross-validation, which helps mitigate background interference and improve diagnostic consistency. However, transitioning these theoretical sensor prototypes into practical, portable in situ diagnostic tools for latent infections involves substantial engineering and validation challenges that require further resolution before widespread field application.

### 6.2. Smart-Responsive Precision Nanodelivery Systems

This is a core direction for resolving the challenges associated with stability and the duration of efficacy. In the short term, by developing “smart” nanocarriers responsive to the pathogenic microenvironment (e.g., pH, temperature, or pathogen-secreted laccases and cellulases), an “on-demand, pathogen-triggered” drug release strategy can be theoretically conceptualized [110]. However, the available field data demonstrating the efficacy of these systems is currently limited, their practical application remains constrained by specific technical limitations. In highly fluctuating field environments, the drug controlled-release of these systems must be rigorously calibrated to match the actual epidemiological development patterns of anthracnose outbreaks [110]. Current platforms struggle with this precise calibration, frequently leading to premature drug leakage or delayed responsiveness. Concurrently, when developing structurally versatile nanocarriers, such as amphiphilic block copolymers, a critical balance must be achieved between field stability and biodegradability [78]. Carriers engineered for extreme stability risk long-term toxic accumulation within agricultural ecosystems. Conversely, overly degradable carriers fail to maintain the required prolonged efficacy. Furthermore, while conceptual paradigms like physical field-assisted nanodelivery (utilizing ultrasound or electric fields) are proposed to theoretically enhance cell membrane permeability and nanoparticle penetration [111], they introduce practical limitations. Integrating such high-energy, equipment-dependent technologies into conventional agricultural machinery cost-effectively, and deploying them across complex field topographies without causing physical damage to host crops, remains an unresolved engineering bottleneck.

### 6.3. Precision Nano-Biopesticides Based on RNAi Technology

Combining the demonstrated delivery efficiency of nanocarriers with the high target specificity of dsRNA offers a potential new pathway for green disease control. By delivering dsRNA/siRNA that specifically silences key genes involved in pathogenicity, growth, appressorium formation, or toxin synthesis (e.g., chitin synthase, melanin biosynthesis genes) in pathogenic fungi, it provides a potential strategy for precise strikes against diseases like anthracnose. However, non-transgenic RNAi strategies require comprehensive evaluation before practical implementation [94]. Specifically, future research must thoroughly investigate the off-target risks associated with the unintentional silencing of homologous genes in beneficial insects, as well as the persistence of dsRNA in soil matrices. Furthermore, understanding the uptake kinetics in non-target organisms is critical. A primary technical hurdle that remains unresolved is the physical delivery barrier posed by the rigid, highly cross-linked melanized cell walls of mature appressoria, which currently severely impedes RNAi efficiency in the pre-penetration phase.

### 6.4. Development of Multi-Functional Nano-Formulations

The development of multi-functional nano-formulations was fundamentally initiated to address the inherent limitations of conventional single-target fungicides, particularly the emergence of pathogen resistance and inadequate field efficacy. The construction of these multi-modal systems aims to co-load chemical fungicides, biocontrol agents, or RNAi reagents onto a single nanoplatform. For example, chitosan-based nanocarriers could potentially be utilized to simultaneously deliver a chemical fungicide (e.g., prochloraz) and dsRNA targeting key genes (e.g., *CsCreA*), exerting synergistic effects at both the chemical inhibition and gene silencing levels. Such a proposed design theoretically possesses the potential to enhance control efficacy and mitigate the development of fungicide resistance.

At the level of direct fungal inhibition, future system design will theoretically no longer rely on traditional chemical toxicity release; instead, it is proposed to focus on utilizing nanomaterials with high surface energy and specific charge properties to potentially disrupt the cell walls/membranes of anthracnose pathogens via electrostatic anchoring. Alternatively, it will aim to target and induce pathological vacuolization, structural shriveling, and melanization precipitation in hyphae and spores, achieving non-specific, multi-site physical destruction at the cellular level. The reduction in the risk of resistance due to this multi-target physical action is a plausible hypothesis, but requires direct experimental confirmation. Nevertheless, a critical unresolved bottleneck lies in achieving strict pathogen specificity; the boundary conditions for such non-specific physical disruption are difficult to calibrate, frequently resulting in unintended collateral phytotoxicity to host tissues during field application. Furthermore, under chronic, sub-lethal exposure to nanomaterials, the potential risk of pathogens evolving novel adaptation mechanisms—such as the upregulation of multi-drug resistance (MDR) efflux pumps or alterations in cell wall composition—remains an unquantified limitation requiring systematic long-term investigation.

At the level of immune amplification and yield enhancement, future research must deeply explore the potential molecular regulatory mechanisms of nanomaterials as immune adjuvants. By precisely triggering a transient burst of ROS in plants and directionally accelerating the host’s phenylpropanoid and flavonoid metabolic pathways, the expression of key disease resistance and development genes (e.g., *PR1*, *COMT*, *PER*) can be upregulated. This is proposed to promote the massive accumulation of defensive secondary metabolites (e.g., quercetin, cinnamaldehyde) and endogenous hormones (e.g., salicylic acid/jasmonic acid (SA/JA)). However, overstimulation often leads to excessive ROS accumulation, inducing severe oxidative stress in plants rather than beneficial defense upregulation. While utilizing metallic building blocks to supplement crops with trace elements (e.g., Cu, Zn, Mn) is proposed to achieve vegetative growth promotion, the fundamental contradiction between short-term nutritional benefits and the long-term ecological risks of heavy metal accumulation in agricultural soils remains a critical barrier preventing these multi-functional systems from becoming fully field-ready.

### 6.5. Green Synthesis Based on Natural Resources and Integrated Postharvest Preservation

Utilizing plant extracts (e.g., polyphenol-rich extracts, essential oils) and microbial metabolites as reducing agents, stabilizing agents, or functionally active components for the “green synthesis” of nanomaterials—or utilizing fruit extracts (e.g., pomegranate peel) to reduce metal ions—offers a potential approach to reducing production costs and enhancing environmental biocompatibility [107]. However, natural extracts are chemically highly complex and vary significantly based on season, geography, and extraction protocols. Consequently, green-synthesized nanomaterials exhibit severe issues with batch-to-batch reproducibility. Furthermore, undefined organic impurities from these extracts can unpredictably interfere with the surface chemistry and targeted release kinetics of the resulting nanoparticles, posing a major barrier to scale-up manufacturing and regulatory standardization.

In the application of postharvest fruit preservation, nanocomposite coatings and packaging materials are advancing toward integrating gas regulation with the sustained release of antimicrobial substances. For instance, combining natural polymers with layered double hydroxides (OLDHs) can enable the long-term controlled release of volatile antimicrobial components, such as plant essential oils. Meanwhile, introducing photocatalytic nanomaterials (e.g., TiO_2_) into composite coatings has demonstrated the potential facilitates the degradation of ethylene in the microenvironment, thereby delaying fruit senescence [107]. To maintain the sensory quality of fruits while inhibiting anthracnose, future coating designs must further optimize their water vapor transmission rate (WVTR) and gas permeability to prevent fruits from undergoing anaerobic respiration due to excessive gas barrier properties.

### 6.6. Elucidation of Multi-Omics Mechanisms and Integration of Green Comprehensive Management Systems

Future fundamental research must rely on multi-omics technologies—including transcriptomics, proteomics, and metabolomics—to systematically elucidate the complex interaction networks among “nanomaterials-pathogens-hosts” and to identify more highly efficient and safe specific molecular targets [78]. However, current multi-omics approaches struggle to capture the highly dynamic, spatiotemporal variations in these interactions under fluctuating field conditions, often resulting in reductionist conclusions that fail to reflect actual in-planta performance. Moreover, the molecular targets identified through in vitro omics screens have rarely been validated through reverse genetics in the context of nanomaterial exposure, leaving a significant gap between correlative observations and causal mechanistic understanding. From a systems-level perspective, the integration of nano-phytosanitary technologies with disease-resistant variety breeding, ecological regulation, and agronomic cultivation management—while frequently proposed—faces unresolved challenges. It remains unclear how nanomaterial-induced plant immune responses interact with genetically encoded resistance pathways, whether chronic nanoparticle exposure could inadvertently compromise beneficial plant–microbe symbioses that underpin ecological regulation strategies, and how nano-formulation application schedules can be rationally aligned with existing agronomic practices without exacerbating off-target ecological effects. These knowledge gaps must be systematically addressed before a truly comprehensive green disease control system can be realized.

### 6.7. Toxicological Safety Assessment, In Vivo Migration Behavior, and Regulatory Frameworks for Nanomaterials

The large-scale application of agricultural nanotechnology is constrained by whole-life-cycle safety assessments and regulatory frameworks. At the ecotoxicological level, there is currently a severe lack of scientific data regarding multi-trophic ecotoxicity. Future research must urgently address the bioaccumulation and biomagnification patterns of inorganic nanoparticles within agricultural food chains, as well as their long-term disruptive impacts on the structural balance of beneficial rhizosphere microbial communities. It is necessary to systematically evaluate the potential toxic effects of long-term nanoparticle exposure on non-target organisms—such as soil microecology, beneficial rhizosphere microbiota, and pollinating insects—to establish environmental safety thresholds. At the plant physiological level, it is essential to elucidate the transmembrane migration and degradation kinetics of nanomaterials (especially formulations containing metal nanoparticles) during root absorption, long-distance transmembrane transport mechanisms within plant vascular bundles, and within fruit tissues, to prevent the potential excessive accumulation of harmful components in edible parts. For postharvest treatments, rigorous sensory and physiological evaluations of moisture loss, browning, and off-flavor generation in nano-coated fruits must be conducted [107]. Ultimately, commercial promotion urgently requires collaboration among multidisciplinary fields and international regulatory agencies to formulate unified risk assessment standards and regulatory frameworks that encompass nanomaterial preparation and characterization, field application protocols, and food residue limits.

## 7. Conclusions

Nanotechnology offers a promising research direction and may, with further development, contribute to the prevention and control of plant anthracnose, driving a potential shift from traditional chemical control toward a multidimensional, integrated physicochemical synergistic management approach. Nanomaterials have shown the capacity to serve not only as active fungicidal ingredients but also as versatile carriers for the targeted delivery and controlled release of pesticides. Current experimental evidence suggests that these materials may act through multiple synergistic pathways. For instance, they have been proposed to block appressorium formation, intervene in the hemibiotrophic-to-necrotrophic lifestyle transition, disrupt the structural integrity of pathogen cell membranes, induce intracellular ROS bursts, and activate plant systemic acquired resistance (SAR). However, it must be emphasized that these represent proposed or experimentally suggested mechanisms rather than established mechanisms of anthracnose control. While these integrated pathways provide a mechanistic basis for exploring interventions against latent *Colletotrichum* infections and may contribute to future resistance-management strategies, current insights are primarily deduced from in vitro observations. Systematic field evidence in actual agricultural ecosystems remains limited, underscoring the need for further practical validation.

Due to this evidence gap, translating this technology from laboratory prototypes to field-scale disease management and postharvest industrial applications is constrained by several critical bottlenecks. Foremost among these is the lack of systematic evaluations regarding the long-term environmental behavior and ecotoxicological effects of nanomaterials within complex agroecosystems and storage/transportation microenvironments. Specifically, the migration and transformation patterns of nano-formulations across multi-phase media such as soil and foliar/fruit surfaces, their transmembrane transport kinetics within plant vascular bundles, the impacts of long-term low-dose exposure on beneficial rhizosphere microbial communities and pollinating insects, as well as the residual degradation dynamics and safety of postharvest coatings on edible fruit surfaces, remain the core constraints hindering their regulatory approval and widespread adoption. Furthermore, the bottleneck of scaling up production economically and the absence of unified global regulatory standards have also delayed the market access of nanopesticides.

Looking ahead, future research priorities should focus on the following key directions. First, establishing a nanopesticide research and development model based on the “Safe-by-Design” principle to integrate environmental compatibility at the source of material design. This involves utilizing *Colletotrichum*-specific secreted enzymes (such as laccases and extracellular hydrolases) or changes in the infection microenvironment as stimuli to ensure precise and controllable smart-responsive, on-demand drug release. Second, relying on integrated multi-omics analysis to deeply decipher the tripartite molecular interaction networks among nanomaterials, pathogens, and host plants. This effort should particularly focus on discovering highly specific antimicrobial targets related to appressorium virulence—such as those disrupting melanin biosynthesis or chitin synthesis—and immune activation nodes. Third, constructing a standardized evaluation system that encompasses the entire lifecycle environmental fate of nano-formulations, food safety residue thresholds, and human health exposure risks, while simultaneously promoting the formulation of internationally coordinated regulatory frameworks. Through the profound cross-disciplinary integration of materials science, plant pathology, and ecotoxicology, nano-phytosanitary technology demonstrates the potential to support a balance among agricultural production safety, crop quality enhancement, and ecosystem health. Ultimately, subject to further comprehensive field and toxicological validation, these advancements may provide a viable and sustainable approach for the management of major fungal diseases such as anthracnose.

## Figures and Tables

**Table 1:** Inhibitory effects and mechanisms of action of metal and metal oxide nanoparticles against *Colletotrichum* species.

Material	Particle Size (nm)	Pathogen	Effective Concentration	Maximum Inhibition Rate/Effect	Mechanism of Action	Experimental Level	Ref.
AuNPs	~10–20	*C. gloeosporioides*	75 mg·L^−1^	Mycelial growth inhibition of 61%, spore germination rate reduced to 25%; disease incidence in detached fruits reduced to 21%.	Disrupts cell membrane integrity and increases membrane permeability; significantly upregulates defense genes (*PDF1.2* by 16-fold, *LOX* by 24-fold) and transcription factors (WRKY33 by 21-fold); enhances antioxidant enzyme activities; increases the uptake of nutrients such as P, N, K, and Zn in roots, leaves, and fruits.	Greenhouse/Detached fruit	[50]
AgNPs	5–24	*C. gloeosporioides*	56 µg·mL^−1^	~90%	Penetrates and disrupts the cell membrane; releases Ag^+^ to catalyze ROS generation; inhibits spore germination and appressorial melanization.	In vitro	[51]
Ag-SiO_2_NPs	118.2 ± 4.6	6 plant pathogenic fungi including *C. gloeosporioides*	0.5–25 µg·mL^−1^	100% mycelial growth inhibition of *C. gloeosporioides* at 25 ppm; 23.5–44.1% inhibition against multiple fungi at 0.5 ppm.	Sustained release of Ag^+^ through the porous SiO_2_ shell; primarily inhibits fungal growth by inducing an ROS burst.	In vitro	[52]
CuNPs	5–10	*C. asianum*, *C. siamense*, etc.	150–300 µg·mL^−1^	100% (Lethal)	EC_50_ values for various mango anthracnose fungi range from 142–187 µg·mL^−1^; effectively inhibits spore germination and appressorium formation.	In vitro	[53]
Cu_2_ONPs	~180	*C. capsici*	70 mg	90%	Achieves highly efficient fungicidal effects leveraging the small-size effect and specific exposed crystal facets generated by its octahedral morphology.	In vitro	[54]
Cu(OH)_2_ NPs	~26.5	*C. siamense*	300 mg·L^−1^	99.16%	Induces ROS burst; causes DNA damage; inhibits superoxide dismutase (SOD) enzyme activity and promotes malondialdehyde (MDA) accumulation.	In vitro	[55]
CuONPs	~46 (TEM) ~62 (DLS)	*C. gloeosporioides*	MIC: 125 µg·mL^−1^	74.69% (at 1000 µg·mL^−1^)	Induces mycelial morphological deformation; triggers excessive ROS accumulation and membrane lipid peroxidation, disrupting the mycelial cell membrane and causing leakage of cellular contents.	In vitro	[56]
ZnONPs	20–70	*Colletotrichum* sp.	15 mmol·L^−1^ (approx. 1220 µg·mL^−1^)	~96%	Mycelial vacuolation; cytoplasmic liquefaction; nanoparticle adsorption leads to structural damage of the mycelia.	In vitro	[57]
MgONPs	20–30	*C. asianum*	800 µg·mL^−1^	100% (Lethal)	Induces ROS; disrupts the cell membrane; inhibits appressorium formation.	In vitro	[58]
PdNPs	200–250	*C. gloeosporioides, F. oxysporum*	50 µg·well^−1^	Inhibition zone diameter against *C. gloeosporioides* reaches up to 3.6 mm (Day 2).	The binding of Pd^2+^ to DNA phosphate groups and the inhibition of key enzymes such as succinate dehydrogenase and creatine kinase.	In vitro	[59]
PtNPs	10–50	*C. acutatum*	4.8 µg·well^−1^	Inhibition zone diameter reaches up to 15 mm.	Green-synthesized using plant gum extracts, exhibiting significant antifungal activity.	In vitro	[60]
Oxovanadium(IV) complexes	18–24	*C. falcatum*, *C. pallescence*	200 µg·mL^−1^	80.6% (*C. falcatum*), 76.0% (*C. pallescence*)	Chelation of the ligand with vanadium ions enhances lipophilicity, promoting penetration through the cell membrane; may interfere with normal cellular processes by interacting with cellular enzyme systems or forming hydrogen bonds.	In vitro	[61]

**Table 2:** Antibacterial efficacy and mechanisms of natural polymer nanomaterials against *Colletotrichum* spp.

Nano- Materials	Active Ingredients	Particle Size (nm)	Tested Pathogens	Effective Concentration	Maximum Inhibition Rate/Effect	Key Mechanisms of Action	Functional Role	Ref.
CSNPs	Chitosan	~30–70	*C. gloeosporioides*	0.5% (*w*/*v*)	97% (Mycelial growth)	Positively charged nanoparticles interact with negatively charged fungal cell membranes, altering membrane permeability; the small size (30 nm) facilitates cell penetration, disrupting intracellular Ca^2+^ homeostasis and leading to cell death.	Intrinsic antifungal agent	[75]
HLDP-CS	Hydrophilic-lipophilic diblock polymer- chitosan	232 ± 20	*C. gloeosporioides*	CS: 5 mg·L^−1^	Colony diameter reduced from 6.2 cm to 5.0 cm; spore germination rate decreased to 4.0%; fruit disease index dropped from 59.26 to 13.58	Destroying fungal cell wall/membrane integrity via electrostatic interactions, inhibiting mycelial growth and spore germination; HLDP acts as an adjuvant to efficiently deliver chitosan, activating plant secondary metabolism (flavonoids, phenylpropanoids) and phytohormone (SA, ABA) pathways, thereby amplifying the immune response.	Carrier- enhanced/Dual-functional system	[78]
CHT- Cu NPs	Chitosan- copper	~150 (DLS)	*C. gloeosporioides*, *C. ciceri*, *F. ciceri*, etc.	200–400 µg·mL^−1^ (Complete inhibition)	100%	The polymer matrix enables the sustained release of Cu^2+^ (continuous release for 192 h); chitosan disrupts membrane surface integrity, while Cu^2+^ enters the cell to interfere with enzyme activity and redox balance, causing cumulative toxicity.	Carrier- enhanced/Dual-functional system	[76]
CS- AgNPs	Chitosan- silver	~4	*C. truncatum*	0.0005% (*w*/*v*)	100% (Spore germination)	The chitosan matrix binds to the cell membrane via electrostatic interactions, increasing membrane permeability; AgNPs attach to the membrane and enter the cell, inhibiting genes related to melanin synthesis (e.g., *LAC1*) and blocking appressorium formation.	Carrier- enhanced/Dual-functional system	[79]
TiNPs + ChNPs/Alg	Alginate- titanium/ chitosan	4.5–5.2	*C. graminicola*	50–70 beads/pot	Complete disease control	Chitosan beads (ChNPs) alone provide 81% antifungal activity; titanium nanoparticles (TiNPs) provide additional chemical/catalytic toxicity; the alginate matrix encapsulates both particles and enables sustained release.	Carrier- enhanced/Dual-functional system	[6]

**Table 3:** Physicochemical properties and antifungal efficacy of chemical fungicide-loaded nano-delivery systems against *Colletotrichum* species.

Nanocarrier	Chemical Active Ingredient	Size & Zeta Potential	Target Pathogen	Efficacy	Ref.
PLA	Azoxystrobin	Size: 130.9 nm; Zeta potential: −23.9 mV	*C. higginsianum*	Smaller particle size led to higher drug release rates and cumulative release and lower LC_50_ values; the uptake efficiency of 130 nm microspheres by hyphae was 7.25 times that of 3078 nm microspheres; smaller microspheres induced higher ROS levels in hyphae and stronger inhibition of SOD and CAT activities.	[83]
CS/O-CMCS	Tebuconazole (TBA)	Size: 173.7 nm; Zeta potential: +35.27 mV	*C. gloeosporioides*	Target enrichment and release of TBA achieved by exploiting fungal affinity for the biocompatible carrier; EC_50_ was 3.13 times that of TBA SC; the contact angle change rate of the loaded formulation on mycelia was 102 times that of the SC, significantly improving target deposition; the prolonged half-life resulting from enhanced foliar adhesion necessitates a continuous evaluation of its long-term safety.	[84]
ZIF-8	Prochloraz (Pro)	Size: 139.6 nm; Zeta potential: +15.0 mV	*C. fructicola*	Loading capacity of 15.94%. 89.8% Pro release within 24 h at pH 5.0; EC_50_ (0.0150 mg·L^−1^) was significantly lower than that of free Pro (0.0429 mg·L^−1^); treatment effectively maintained fruit quality indicators such as firmness, weight, and sugar content; the formulation could be systemically transported in strawberry plants after foliar or root application.	[85]
MSNs-CMC-TPP	Pyraclostrobin (Py)	Size: ~136 nm; Zeta potential: −103 mV	*C. orbiculare*	Py loading capacity of 32%; drug release persisted for over 14 days; pH-responsive release mechanism combined with TPP-mediated mitochondrial targeting for enhanced efficacy; low-concentration efficacy surpassed commercial Py SC, demonstrating high eco-safety and pesticide-reducing potential.	[86]
MSNs-β-glucans	Chlorothalonil (CHT)	Size: 27.5–39 nm; Zeta potential: N/A	*C. truncatum*	CHT loading content of 23.01%; EC_50_ values against various pathogens were lower than those of commercial CHT WP formulation; the LC_50_ for earthworms was 2.25 times that of the WP, and the 96 h LC_50_ for zebrafish was 2.66 times that of the WP, demonstrating superior biosafety and low non-target toxicity; CHT release triggered by pathogen-secreted β-glucanase.	[87]
rGO-Cu_2−x_Se	Captan(CP)	Size: 514–588 nm; Zeta potential: +9 mV	*C. capsici*	Loading capacity of 26%, encapsulation efficiency of 36%. Cumulative CP release reached 66% under simulated fungal acidic conditions (pH 5.5), compared to only 30% at neutral pH (pH 7.2); foliar retention of CP increased from 35% to 65% after loading; CP leaching loss decreased from 70% to 26% after loading, minimizing environmental pollution and Cu-accumulation toxicity; in acidic soil, increasing ionic strength further suppressed CP release; the MIC_50_ of the formulation at pH 5.5 was 81 µg·mL^−1^, significantly lower than that of free CP (160 µg·mL^−1^); in chili plants, the disease index at 5 days post-treatment was reduced from 37% (water control) to 16%, outperforming the CP emulsion (22%).	[5]
CXP-NH_3_	Trifloxystrobin (Tri)	Size: 311–573 nm; Zeta potential: +50.3 to +60.7 mV	*Colletotrichum* spp.	Drug loading content of Tri could be regulated by side-chain modification of the carrier; significantly delayed the degradation of Tri under 254/302 nm UV light; significantly improved rainfastness of Tri on leaves. Under simulated 36 mm rainfall, the drug retention rate increased by over 44%; loading significantly enhanced the inhibitory effect of Tri on spore germination and mycelial growth of *Colletotrichum* and *Pyricularia* species and weakened the penetration ability of the pathogens.	[88]
Cu-MOF	Difenoconazole (DIF)	Size: ~408.66 nm; Zeta potential: −15.8 mV	*C. gloeosporioides*	DIF loading content of 19.18%; DIF release under NIR irradiation was 2.7 times higher than that under dark conditions; DIF retention on hydrophobic/hydrophilic leaves was 9.4 and 36.5 times higher than that of the ME formulation, respectively; efficient uptake by fungal hyphae; the control effect after 14 days (43.4%) was significantly higher than that of DIF ME (24.8%).	[89]

**Table 4:** Comparative analysis of different nanomaterial classes against *colletotrichum*.

Nanomaterial Class	Effectiveness & Experimental Validation	Main Advantages	Main Limitations	Most Promising Practical Applications	Environmental Risks	Technology Readiness Level
Metal & Metal Oxide NPs	High effectiveness. Validated mostly in vitro, with limited greenhouse/field trials.	Potent, rapid broad-spectrum biocidal activity; disrupts multiple cellular targets simultaneously.	High potential for phytotoxicity; dose-dependent toxicity; non-biodegradable.	Low-dose field sprays; integrated into slow-release core–shell matrices to minimize soil loading.	Very High (Non-biodegradable; accumulates in soil/plants; toxic to PGPR and aquatic life)	Moderate (mostly in vitro, some field trials)
Natural Polymer NPs	Moderate to High effectiveness. Extensively validated in vitro, greenhouse, and post-harvest in vivo trials.	Highly biocompatible; biodegradable; acts as plant immune elicitors; excellent film-forming ability.	Efficacy depends heavily on environmental pH and molecular weight; lower acute toxicity compared to metals.	Post-harvest edible coatings for fruit preservation; carrier matrices for biopesticides.	Low (Highly biodegradable; minimal ecological footprint)	High (widely tested in field and postharvest)
Carbon-Based NPs	Moderate effectiveness. Validation is strictly confined to in vitro and detached-fruit laboratory tests.	Ultra-high specific surface area; good physicochemical stability; capable of modulating rhizosphere ecology.	Complex and costly synthesis; highly variable degradation rates; potential long-term accumulation.	Soil amendments/rhizosphere conditioners; functional UV-blocking fillers in composite films.	Moderate to High (variable soil degradation rates; unknown food chain fate)	Low (primarily laboratory-confined)
Nano-Delivery Systems	Very High effectiveness. Validated in vitro, greenhouse, and increasingly in open-field simulated washout tests.	Enables targeted, smart-responsive release; protects active ingredients from UV/rain wash-off; enhances bioavailability.	High manufacturing costs; formulation stability issues; complex regulatory hurdles.	Delivery of easily degraded biopesticides (e.g., essential oils, dsRNA) and targeted chemical fungicide reduction.	Variable, depends on the carrier matrix (synthetic vs. natural) and the loaded active ingredient.	Moderate to High (some polymer hybrids are field-tested, while MOFs remain in lab stage)

## Data Availability

The data are available from the corresponding authors.
